# Diagnostic value of digital clock drawing test in comparison with CERAD neuropsychological battery total score for discrimination of patients in the early course of Alzheimer’s disease from healthy individuals

**DOI:** 10.1038/s41598-019-40010-0

**Published:** 2019-03-05

**Authors:** Stephan Müller, Laura Herde, Oliver Preische, Anja Zeller, Petra Heymann, Sibylle Robens, Ulrich Elbing, Christoph Laske

**Affiliations:** 1 Department of Psychiatry and Psychotherapy, Eberhard Karls University, Tübingen, Germany; 2 Geriatric Center at the University Hospital, Eberhard Karls University, Tübingen, Germany; 3 University Witten/Herdecke, Department of Psychology and Psychotherapy, Witten, Germany; 4 Nuertingen-Geislingen University (HfWU), Institute of Research and Development in Art Therapies, Nuertingen, Germany; 5 German Center for Neurodegenerative Diseases (DZNE), Tübingen, Germany; 6 Section for Dementia Research, Hertie Institute for Clinical Brain Research and Department of Psychiatry and Psychotherapy, Eberhard Karls University, Tübingen, Germany

## Abstract

The early detection of cognitive impairment or dementia is in the focus of current research as the amount of cognitively impaired individuals will rise intensely in the next decades due to aging population worldwide. Currently available diagnostic tools to detect mild cognitive impairment (MCI) or dementia are time-consuming, invasive or expensive and not suitable for wide application as required by the high number of people at risk. Thus, a fast, simple and sensitive test is urgently needed to enable an accurate detection of people with cognitive dysfunction and dementia in the earlier stages to initiate specific diagnostic and therapeutic interventions. We examined digital Clock Drawing Test (dCDT) kinematics for their clinical utility in differentiating patients with amnestic MCI (aMCI) or mild Alzheimer’s dementia (mAD) from healthy controls (HCs) and compared it with the diagnostic value of the Consortium to Establish a Registry for Alzheimer’s Disease (CERAD) neuropsychological battery total score. Data of 381 participants (138 patients with aMCI, 106 patients with mAD and 137 HCs) was analyzed in the present study. All participants performed the clock drawing test (CDT) on a tablet computer and underwent the CERAD test battery and depression screening. CERAD total scores were calculated by subtest summation, excluding MMSE scores. All tablet variables (i.e. time in air, time on surface, total time, velocity, pressure, pressure/velocity relation, strokes per minute, time not painting, pen-up stroke length, pen-up/pen-down relation, and CDT score) during dCDT performance were entered in a forward stepwise logistic regression model to assess, which parameters best discriminated between aMCI or mAD and HC. Receiver operating characteristics (ROC) curves were constructed to visualize the specificity in relation to the sensitivity of dCDT variables against CERAD total scores in categorizing the diagnostic groups. dCDT variables provided a slightly better diagnostic accuracy of 81.5% for discrimination of aMCI from HCs than using CERAD total score (accuracy 77.5%). In aMCI patients with normal CDT scores, both dCDT (accuracy 78.0%) and CERAD total scores (accuracy 76.0%) were equally accurate in discriminating against HCs. Finally, in differentiating patients with mAD from healthy individuals, accuracy of both dCDT (93.0%) and CERAD total scores (92.3%) was excellent. Our findings suggest that dCDT is a suitable screening tool to identify early cognitive dysfunction. Its performance is comparable with the time-consuming established psychometric measure (CERAD test battery).

## Introduction

The early detection of cognitive dysfunction or dementia will become one of the major challenges with respect to the increasing number of cognitively impaired individuals due to aging population worldwide. Additionally, there is a significant delay in the diagnosis of dementia, which may limit the effectiveness of pharmacological interventions. Every second dementia case is even not detected^1–5^. Clinicians and healthcare systems are confronted with limited financial and medical resources demanding reasonable, cost-effective clinical methods that combine diagnostic accuracy and effectiveness. This challenge is crucial for AD as it is expected that the number of people affected will increase significantly in the near future^6^.

It is generally accepted that mild cognitive impairment (MCI) is an intermediate phase located in a continuum between normal cognitive aging and dementia due to Alzheimer’s disease (AD)^7,8^. Especially individuals diagnosed with amnestic MCI (aMCI) show a significantly increased annual risk of conversion to AD compared to others in their age group^7^.

At present, diagnostic methods of MCI or dementia are time-consuming (psychometric testing), invasive (cerebrospinal fluid examination [CSF]) or expensive (neuro-imaging) and not suitable for wide application as required by the high number of people at risk. For example, criterion-standard neuropsychological testing (e.g. CERAD neuropsychological test battery) for all individuals with high risk of contracting the disease could objectify cognitive malfunction in a variety of cognitive parameters, but would be expensive, time consuming, and an expertise is needed. Furthermore, a detailed neuropsychological assessment might be unreasonable for some individuals^9^.

Thus, a screening instrument is largely needed enabling to detect individuals with cognitive dysfunction and dementia at an early stage to suggest advanced diagnostic procedures with CSF examination and neuro-imaging in the presence of positive screening outcomes. Such a screening test should be sensitive, quick and easy to perform, affordable, and should avoid invasive procedures. In addition scoring and test score interpretation should be delegable to clinical assistants in a broad clinical setting.

Drawing and writing require a variety of cognitive skills challenging visual perception and encoding, attention, anticipatory thinking, motor planning and executive functions^10–12^. These demands suggest that it might be vulnerable to cognitive dysfunction and thus the accurate assessment of visuo-constructive abilities might facilitate the detection of such disabilities^13^. However, in general only the final result of the drawing is included in the evaluation, important parameters such as time to complete the task, stroke length, pressure, velocity, intermissions, in-air or on-surface trajectories needed to perform each task are not collected. Additionally, the proportion of patients worried of constructional deficits is very low. In general, patients and their family members usually seek medical attention for memory or other behavioral concerns^14^.

The use of the digitized devices provides the unique possibility to capture the entire sequence during visuo-constructional task performance and recent research using this approach has demonstrated its worthiness in differentiating individuals in the course of AD^15–17^.

Preliminary results of a newly developed tablet-based screening test using movement kinematics while drawing a three-dimensional house demonstrated highest rates of sensitivity (80.0%) but poor specificity (65.0%) when time in air (i.e. time while moving the pen from one stroke to the next) was used to differ between individuals with amnestic MCI (aMCI) and healthy controls (HCs). In discriminating patients with mild dementia due to AD (mAD) from healthy subjects, time in air revealed excellent sensitivity (i.e. 80.0%) and specificity (i.e. 85.0%)^16^. In this previous work three variables, namely pen-up time (i.e. time in air), pen-down time (i.e. time the pen is touching the surface while drawing), and the summation of both variables (i.e. total time) were separately captured and analyzed with regard to their contribution in differentiating between the healthy subjects and the patient groups. In contrast to time in air, the other variables (i.e. total time and time on surface) did not reach satisfactory accuracy values. As a limitation, the variables that have been assessed during house-drawing have not been analyzed with respect to its discriminative power comparing the patient groups (i.e. aMCI and mAD). Additionally, in this early approach the software’s ability to capture a variety of variables during the drawing process has not been fully exploited.

In a subsequent study, the three variables (i.e. time in air, time on surface, and total time) were again used to examine their significance during Clock Drawing Test (CDT) that was implemented on a tablet. This tablet-based CDT offered the possibility to capture these variables digitally (dCDT) while patients with aMCI, mAD or healthy controls performed the task and were scored according to Shulman^18^. Comparable to the results of the previous study using a three-dimensional house, only time in air was able to discriminate patients with aMCI from healthy subjects at an acceptable level with an accuracy of 78.0%. Surprisingly, even patients with normal CDT scores could be differentiated from healthy subjects with 79.5% accuracy. With an accuracy of 87.2%, time in air revealed a comparable screening value as the conventional paper-pencil based CDT (i.e. 87.1%) in discriminating patients with mAD from healthy subjects^15^.

In the present study, we examined the diagnostic value of dCDT variables in discriminating aMCI or mAD patients from HCs and compared it with the discriminative validity of the Consortium to Establish a Registry for Alzheimer’s Disease (CERAD) neuropsychological battery total score^19^. The CERAD total score has shown its diagnostic accuracy in discriminating HC individuals from patients with MCI, or AD^19^. However, administration of this comprehensive test-battery takes at least 45 minutes and an expert is necessary for the implementation, evaluation and interpretation of the test-scores.

In contrast to our previous studies, we now use the full functionality of the tablet software to capture a variety of variables during dCDT with the aim to compete against the CERAD total score. In addition to variables already used in our earlier studies (i.e. time in air, time on surface, and total time) we assessed velocity (i.e. drawing speed), pressure, pressure/velocity relation, strokes per minute, time not painting (i.e. intermission), pen-up stroke length (i.e. in-air distance while moving the pen from one stroke to the next) and pen-up/pen-down relation in order to raise the diagnostic accuracy to the level of an established test battery or even beyond.

We hypothesize that, compared to the previous approach, where only one of three available variables was considered (i.e. time in air OR time on surface OR total time), the inclusion of all available variables mentioned above or their specific combination might result in a stronger predictive value compared to the CERAD total score.

## Materials

### Participants

A total of 381 right-handed^20^ individuals (193 females and 188 males) with a mean age of 70.6 ± 8.2 years were included. All participants had normal hearing and normal or corrected-to-normal vision. All individuals were able to perform the neuropsychological assessment and the drawing task. None of them were physically handicapped that might interfere with his or her ability to complete the assessments. There were no other neurological or psychiatric disorders elicited that was unrelated to his or her diagnosis. The absence of depressive symptoms was assessed using the German 15-item version of the Geriatric Depression Scale (GDS; exclusion criterion GDS > 5)^21^. The local ethical committee at the University Hospital of Tübingen approved the study. All methods were performed in accordance with the relevant guidelines and regulations. An informed consent form was signed by all individuals after receiving a detailed explanation of the study.

### Patients with aMCI or mAD

Patients with aMCI (n = 138) or mAD (n = 106) were recruited from the Memory Clinic of the Department of Psychiatry and Psychotherapy at the University Hospital of Tübingen. All subjects underwent diagnostic work-up for dementia including physical, neurological, and psychiatric examinations as well as brain imaging. Routine laboratory tests included Lues serology and analysis of vitamin B12, folic acid, and thyroid-stimulating hormone levels.

According to current criteria, patients with aMCI did not show signs of probable AD but revealed verbal or visual episodic memory deficits, that did not interfere with activities of daily living^8,22,23^.

The diagnostic criteria for mAD were defined according to the National Institute of Neurological and Communicative Disorders and Stroke Alzheimer’s Disease and Related Disorders Association^22,24^. All of patients with mAD had a score of 4 on the Global Deterioration Scale^25^.

### Healthy control group

Confirmed by a clinical interview, HC individuals (n = 137) did not reveal cognitive disturbances or show any signs of neurological or psychiatric disorders.

## Methods

### Neuropsychological assessment

All participants were confronted with the modified German version of the CERAD neuropsychological test battery including the Mini-Mental state examination (MMSE)^26–28^. CERAD total scores were calculated by subtest summation without MMSE scores according to the method described by Chandler *et al*.^29^.

### Digital clock drawing test

All participants had to complete the CDT on the tablet using a digital pen on a Microsoft Surface Pro 4 digitizer. They were prompted to draw a clock face and to write all numbers on the correct position and to indicate “10 past 11 o’clock” using hands. According to the Shulman criteria results were scored from 1 point (i.e. a perfectly accomplished CDT) to 6 points (i.e. severe impairment; no recognizable clock). A score ≥3 was considered as impaired^30^. Movements were sampled at a frequency of 120 Hz with a spatial accuracy of 0.25 mm. Besides *time in air* (i.e. pen-up state; time while moving the pen from one stroke to the next), *time on surface* (i.e. pen-down state), and *total time* (i.e. time in air plus time on surface) we assessed *velocity* (i.e. drawing speed), *pressure*, *pressure/velocity relation*, *strokes per minute*, *time not painting* (i.e. intermission), *pen-up stroke length* (i.e. in-air distance while moving the pen from one stroke to the next) and *pen-up/pen-down relation*.

### Data analysis

All statistical analyses were run using JMP®, Version 13.1.0, SAS Institute Inc., and p-values < 0.05 were considered to be significant. Homoscedasticity was examined using Levene’s test. The Pearson chi-square test was applied to assess group differences in gender distribution, group differences in CDT and GDS scores were examined running the nonparametric Kruskal-Wallis test. Between-group comparisons of age, education, global cognition (MMSE), CERAD total scores, and tablet parameters, were conducted with one-way analysis of variance (ANOVA) using the group (Control, MCI and mAD) as between-subject factor. Paired comparisons were performed with Tukey adjusted post hoc tests. Between-group comparisons in dCDT variables were Bonferroni adjusted to control for the family-wise error rate.

Combinations of dCDT tablet parameters in addition with CDT scores were entered in an age, educational level and gender adjusted logistic regression model using a forward stepwise inclusion mode. Logistic regression was executed with aMCI against HCs or mAD as dichotomous dependent variable and all tablet parameters as continuous independent variables.

Receiver operating characteristic (ROC) curves of the logistic models and areas under curves (AUCs) were calculated.

To examine the diagnostic value of the model selected variables in differentiating HCs and patients with aMCI or mAD logistic regression analyzes were conducted, each with diagnostic group (i.e. HC vs. aMCI; HC vs. mAD, or aMCI vs. mAD) as the dependent variable and dCDT model selected variables or CERAD total scores as the independent variables respectively. All logistic regression models were adjusted for age, education level and gender.

To evaluate the diagnostic accuracy of dCDT variables compared to CERAD total scores in subjects with normal CDT-score, the same analyses as described above have been executed for subjects with aMCI and no pathological CDT score and HCs.

## Results

### Clinical, neuropsychological and demographic characteristics of the participants

Healthy control individuals, patients with aMCI, or mAD were comparable regarding age, education, GDS scores, and gender distribution. Overall, CDT performance differed significantly between the groups (χ² [8] = 253.69; *p* < 0.0001; Table 1).

**Table 1:** Clinical, neuropsychological, and demographic characteristics of healthy control individuals (HC), patients with amnestic mild cognitive impairment (aMCI) and patients with mild dementia due to AD (mAD).

	Group			p-value
	HC	aMCI	mAD	
	n = 137	n = 138	n = 106	
Age in years	69.6 (7.8)	70.8 (8.4)	71.4 (8.4)	0.177
Years of education	13.2 (2.7)	12.6 (2.9)	12.6 (2.6)	0.169
Gender (F[%]/M[%])	60[44]/77[56]	66[48]/72[52]	55[52]/51[48]	0.454
GDS	2.7 (2.6)	2.9 (2.6)	2.9 (2.4)	0.687
MMSE	29.1 (0.9)	26.5 (1.7)	23.2 (2.4)	<0.0001
CERAD total score	83.6 (9.5)	68.6 (11.0)	52.1 (11.4)	<0.0001
dCDT	1.2 (0.6)	2.1 (0.8)	2.9 (0.8)	<0.0001
CDT Score 1; n(%)	109 (80.7)	37 (26.8)	7 (6.6)	
CDT Score 2; n(%)	28 (19.3)	58 (42.0)	12 (11.3)	
CDT Score 3; n(%)	0 (0.0)	43 (31.2)	63 (59.4	
CDT Score 4; n(%)	0 (0.0)	0 (0.0)	24 (22.6)	

Values are displayed in mean (standard deviation); n = number; HC: healthy control individuals; aMCI: amnestic mild cognitive impairment; mAD: mild Alzheimer-type dementia; F[%]/M[%] = number and percentage of female/male; GDS = Geriatric Depression Scale (a higher score indicates more severe depressive symptoms; maximum 15; scores of >5 indicates depression); MMSE = Mini Mental State Examination; CERAD = Consortium to Establish a Registry for Alzheimer’s Disease neuropsychological battery; dCDT = digital Clock Drawing Test (a score ≥3 is considered as impaired).

Patients with mAD revealed significantly greater impairment as indicated by higher CDT scores compared to aMCI (p < 0.0001) and healthy individuals (p < 0.0001). The aMCI group showed significantly more disturbances in CDT than healthy individuals (p < 0.0001).

MMSE scores differed significantly between the investigated groups (F[2,378] = 333.681; p < 0.0001; Table 1). Higher MMSE scores were found in HC individuals compared to aMCI and mAD patients (HC: 29.1 ± 0.9, aMCI: 26.5 ± 1.7; mAD: 23.2 ± 2.4; all p < 0.0001). Better MMSE performance was found in aMCI patients compared to mAD subjects (p < 0.0001).

CERAD total score examination revealed significant differences between HCs, aMCI, and mAD (F[2,378] = 224.474; p < 0.0001; Table 1). HC individuals revealed higher CERAD total scores compared to patients with aMCI or mAD whereas individuals with aMCI scored higher than mAD patients (HC: 83.6 ± 9.5; aMCI: 68.6 ± 11.0; mAD: 52.1 ± 11.1; all p < 0.0001).

Subgroup analyses between HCs and patients with aMCI and normal CDT scores did not show significant differences in demographic variables (i.e. age, education, GDS, and gender distribution). MMSE scores were significantly higher in healthy individuals compared to aMCI patients (HC: 29.1 ± 0.9; aMCI: 26.7 ± 1.9; p < 0.0001), higher CERAD total scores were found in HCs compared to the aMCI group (HC: 83.6 ± 9.5; aMCI: 68.9 ± 10.8; p < 0.0001; Table 2).

**Table 2:** Clinical, neuropsychological, and demographic characteristics of healthy control individuals (HC) and patients with amnestic mild cognitive impairment (aMCI) with normal clock drawing scores (i.e. a score of 1 or 2).

	Group		p-value
	HC	aMCI	
	n = 137	n = 95	
Age in years	69.6 (7.8)	69.6 (8.7)	0.967
Years of education	13.2 (2.7)	12.9 (2.9)	0.622
Gender (F[%]/M[%])	60[44]/77[56]	42[44]/53[56]	0.950
GDS	2.7 (2.6)	2.8 (2.8)	0.756
MMSE	29.1 (0.9)	26.7 (1.9)	<0.0001
CERAD total score	83.6 (9.5)	68.9 (10.8)	<0.0001

Values are displayed in mean (standard deviation); n = number; HC: healthy control individuals; aMCI: amnestic mild cognitive impairment; F[%]/M[%] = number and percentage of female/male; GDS = Geriatric Depression Scale (a higher score indicates more severe depressive symptoms; maximum 15; scores of >5 indicates depression); MMSE = Mini Mental State Examination; CERAD = Consortium to Establish a Registry for Alzheimer’s Disease neuropsychological battery.

### Digital clock drawing performance in healthy individuals, patients with aMCI or mAD

Analysis of dCDT variables found highly significant differences between the investigated groups in total time, time on surface, time in air, pen-up stroke length, strokes per minute, time not painting, pen-up/pen-down relation, velocity, and pressure-velocity relation (all *p* < 0.0001; Table 3). Pressure (p = 0.033) was comparable between groups with according to Bonferroni adjustment. Between-group differences are presented in Table 3.

**Table 3:** Performance on digital clock drawing task variables in healthy control (HC) individuals, patients with amnestic mild cognitive impairment (aMCI) and patients with mild dementia due to AD (mAD).

	Group			p-value
	HC	aMCI	mAD	
	n = 137	n = 138	n = 106	
dCDT total time (ms)	43393.4 (15218.3)	69083.0 (32273.6)	105350.8 (61313.0)	<0.0001 HC < MCI*** HC < mAD*** MCI < mAD***
dCDT time on surface (ms)	14550.9 (5069.5)	18641.4 (6660.3)	21027.8 (7277.5)	<0.0001 HC < MCI*** HC < mAD*** MCI < mAD**
dCDT strokes per minute	39.4 (14.4)	32.7 (12.7)	24.6 (12.1)	<0.0001 HC < MCI*** HC < mAD*** MCI < mAD***
dCDT time in air (ms)	13472.6 (4936.3)	24819.4 (13790.4)	38409.9 (24607.6)	<0.0001 HC < MCI*** HC < mAD*** MCI < mAD***
dCDT time not painting (ms)	34264.4 (19458.7)	52519.8 (38932.7)	79341.5 (59763.3)	<0.0001 HC < MCI*** HC < mAD*** MCI < mAD***
dCDT pen-up stroke length	2189.9 (10358.1)	3001.9 (1633.1)	3612.9 (2462.3)	<0.0001 HC < MCI*** HC < mAD*** MCI < mAD*
dCDT pen-up/pen-down relation	1.07 (0.43)	1.32 (0.69)	1.63 (0.87)	<0.0001 HC < MCI** HC < mAD*** MCI < mAD**
dCDT velocity	9.4 (2.2)	8.3 (2.2)	7.0 (1.9)	<0.0001 HC > MCI*** HC > mAD*** MCI > mAD***
dCDT pressure	0.31 (0.09)	0.29 (0.09)	0.33 (0.12)	0.033
dCDT pressure-velocity relation	2.18 (0.79)	2.21 (1.00)	2.82 (1.38)	<0.0001 HC < mAD*** MCI < mAD***

*p < 0.05; **p < 0.001; ***p < 0.0001.

Values are displayed in mean (standard deviation); n = number; HC: healthy control individuals; aMCI: amnestic mild cognitive impairment; mAD: mild Alzheimer-type dementia; dCDT = digital Clock Drawing Test; ms = time in milliseconds.

Between healthy individuals and aMCI patients who succeed in CDT, significant differences in dCDT parameters were found in total time, time on surface, strokes per minute, time in air, time not painting, pen-up stroke length, and velocity (all p < 0.0014). Pen-up/pen-down relation, pressure, and pressure-velocity relation were comparable between the groups. Between-group differences are presented in Table 4.

**Table 4:** Performance on digital clock drawing task variables in healthy control (HC) individuals and patients with amnestic mild cognitive impairment (aMCI) with normal clock drawing scores (i.e. a score of 1 or 2).

	Group		p-value
	HC	aMCI	
	n = 137	n = 95	
dCDT total time (ms)	43393.4 (15218.3)	64974.2 (30708.4)	<0.0001
dCDT time on surface (ms)	14550.9 (5069.5)	18437.0 (6693.8)	<0.0001
dCDT strokes per minute	39.4 (14.4)	33.5 (12.3)	0.0014
dCDT time in air (ms)	13472.6 (4936.3)	22674.6 (11939.1)	<0.0001
dCDT time not painting (ms)	34264.4 (19458.7)	49038.6 (38198.5)	<0.0001
dCDT pen-up stroke length	2189.9 (10358.1)	2858.9 (1469.1)	<0.0001
dCDT pen-up/pen-down relation	1.07 (0.43)	1.24 (0.65)	0.0223 (ns)
dCDT velocity	9.4 (2.2)	8.4 (2.2)	0.0006
dCDT pressure	0.31 (0.09)	0.29 (0.09)	0.2868
dCDT pressure-velocity relation	2.18 (0.79)	2.15 (0.98)	0.984

Values are displayed in mean (standard deviation); n = number; HC: healthy control individuals; aMCI: amnestic mild cognitive impairment; dCDT = digital Clock Drawing Test; ms = time in milliseconds.

### Predictive value of the digital clock drawing task and CERAD total scores

**Figure 1:**
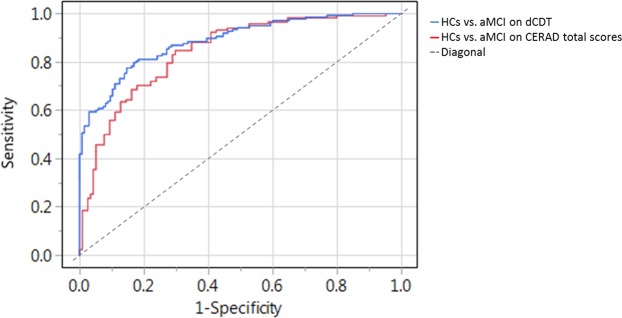
AUCs for HCs against aMCI using dCDT Model a (CDT score, time in air, and time not painting; blue curve) or CERAD total scores (red curve).

Predicting patients with aMCI against healthy individuals, the hybrid of time in air, time not painting, and CDT score (Table 5, Model a) classified 81.5% of the cases correctly (area under the ROC-curve [AUC]: 0.888; p < 0.0001; Fig. 1; Table 6). Using CERAD total score of 80.0 to differentiate between aMCI patients and HCs (AUC: 0.852; p < 0.0001; Fig. 1; Table 5, Model a;) revealed an accuracy of 77.5% (Table 6).

**Table 5:** Diagnostic value of dCDT parameters and CERAD total scores in differentiating healthy individuals (HC) and patients with amnestic mild cognitive impairment (aMCI).

Model	ROC AUC	Selected Variable	Beta (β)	OR	95% CI (OR)		p-value
					Lower	Upper	
a) HC vs. aMCI	0.888	CDT score	1.864	6.452	3.671	11.340	<0.001
		time in air	0.131	1.140	1.081	1.201	<0.001
		time not painting	0.013	1.013	1.000	1.026	0.045
	0.852	CERAD total score (cut-off 80.0 points)	0.137	0.872	0.842	0.903	<0.001
b) HC vs. aMCI with normal CDT scores	0.837	time in air	0.163	1.177	1.117	1.240	<0.001
	0.848	CERAD total score (cut-off 80.0 points)	0.133	0.875	0.843	0.908	<0.001
c) HC vs. mAD	0.973	time in air	0.113	1.120	1.040	1.206	0.003
		CDT score	2.554	12.860	5.892	28.068	<0.001
	0.976	CERAD total score (cut-off 66.0 points)	0.225	0.799	0.750	0.851	<0.001
d) aMCI vs. mAD	0.852	total time	0.008	1.008	1.000	1.016	0.46
		pressure/velocity relation	0.407	1.503	1.120	2.017	0.007
		strokes per minute	−0.028	0.973	0.946	1.000	0.048
		CDT scores	1.298	3.661	2.347	5.710	<0.001
	0.846	CERAD total score (cut-off 58.0 points)	0.126	0.882	0.852	0.913	<0.001

HC: healthy control individuals; aMCI: amnestic mild cognitive impairment; CDT: Clock Drawing Test; CERAD = Consortium to Establish a Registry for Alzheimer’s Disease neuropsychological battery. ROC = Receiver operating characteristics (ROC) curves; AUC = Area under the ROC curve; ß = Logistic regression coefficient, OR = Odds ratio, CI(OR) = Confidence Interval of odds ratio.

**Table 6:** Diagnostic value of dCDT parameters and CERAD total scores in differentiating healthy individuals (HC) and patients with amnestic mild cognitive impairment (aMCI).

	HC vs. aMCI			HC vs. aMCI with normal CDT scores		
	Sensitivity	Specificity	Accuracy	Sensitivity	Specificity	Accuracy
dCDT	85.4^a^	77.5^a^	81.5^a^	81.8^b^	72.6^b^	78.0^b^
CERAD total score	84.6^c^	70.3^c^	77.5^c^	84.5^d^	69.8^d^	76.0^d^

HC: healthy control individuals; aMCI: amnestic mild cognitive impairment; dCDT: digital Clock Drawing Test; dCDT variables in the regression model: ^a^CDT score, time in air, and time not painting; ^b^time in air; CERAD = Consortium to Establish a Registry for Alzheimer’s Disease neuropsychological battery. ^c,d^CERAD total score cut-off 80.0 points.

**Figure 2:**
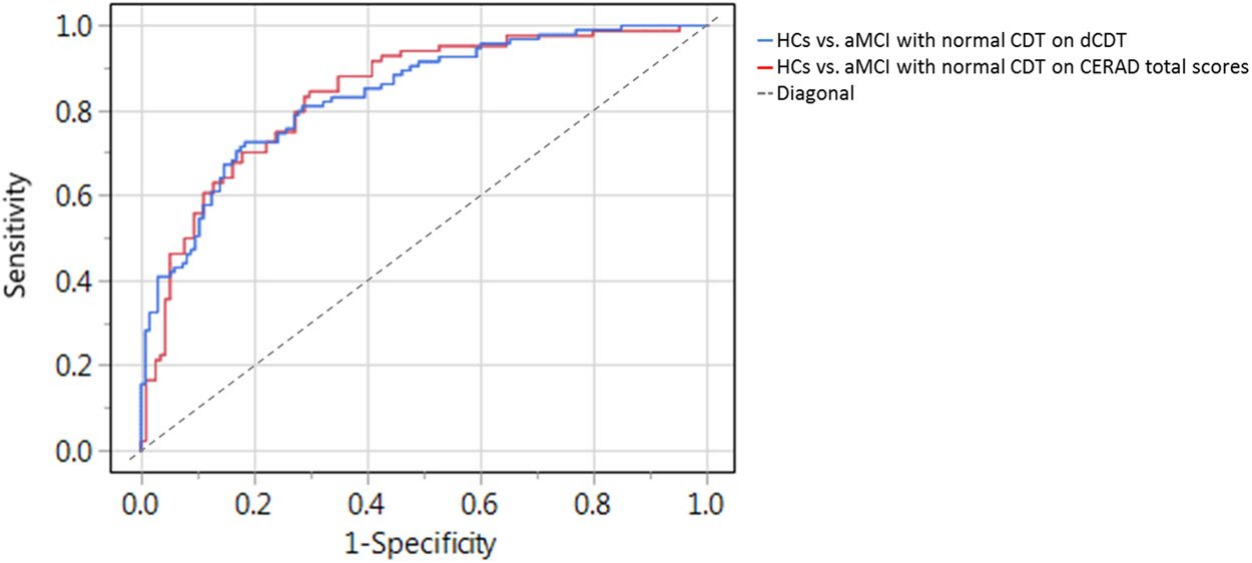
AUCs for HCs against aMCI with normal CDT scores using dCDT Model b (time in air; blue curve) or CERAD total scores (red curve).

Discriminating aMCI with normal CDT scores from HCs (Table 5, Model b) time in air succeeds with an accuracy of 78.0% (AUC: 0.837; p < 0.0001; Fig. 2; Table 6). In discriminating aMCI patients with normal CDT scores from HCs (Table 5, Model b) a CERAD total score of 80.0 revealed an accuracy of 76.0% (AUC: 0.848; p < 0.0001; Fig. 2; Table 6).

**Figure 3:**
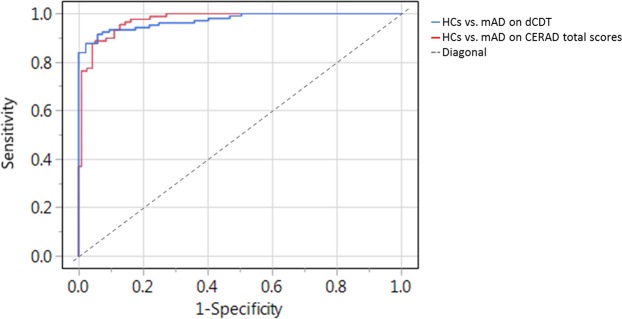
AUCs for HCs against mAD using dCDT Model c (time in air and CDT score; blue curve) or CERAD total scores (red curve).

Using the combination of time in air and CDT score 93.0% of HCs and mAD patients (Table 5, Model c) were classified correctly (AUC: 0.973; p < 0.0001; Fig. 3; Table 7). Between the mAD group and HCs (Table 5, Model c), a total score of 66.0 in the CERAD test battery (AUC: 0.976; p < 0.0001; Fig. 3) revealed an accuracy of 92.3% (Table 7).

**Table 7:** Diagnostic value of dCDT parameters and CERAD total scores in differentiating healthy individuals (HC), patients with amnestic mild cognitive impairment (aMCI) or mild dementia due to Alzheimer’s disease (mAD).

	HC vs. mAD			aMCI vs. mAD		
	Sensitivity	Specificity	Accuracy	Sensitivity	Specificity	Accuracy
dCDT	91.5^a^	94.2^a^	93.0^a^	69.8^b^	89.9^b^	81.2^b^
CERAD total score	88.7^c^	94.9^c^	92.3^c^	76.4^d^	81.4^d^	79.2^d^

HC: healthy control individuals; aMCI: amnestic mild cognitive impairment; mAD: mild dementia due to Alzheimer’s disease; dCDT: digital Clock Drawing Test; dCDT variables in the regression model: ^a^Time in air and CDT score; ^b^total time, pressure/velocity relation, strokes per minute, and CDT scores; CERAD = Consortium to Establish a Registry for Alzheimer’s Disease neuropsychological battery. ^c^CERAD total score cut-off 66.0 points; ^d^CERAD total score cut-off 58.0.

**Figure 4:**
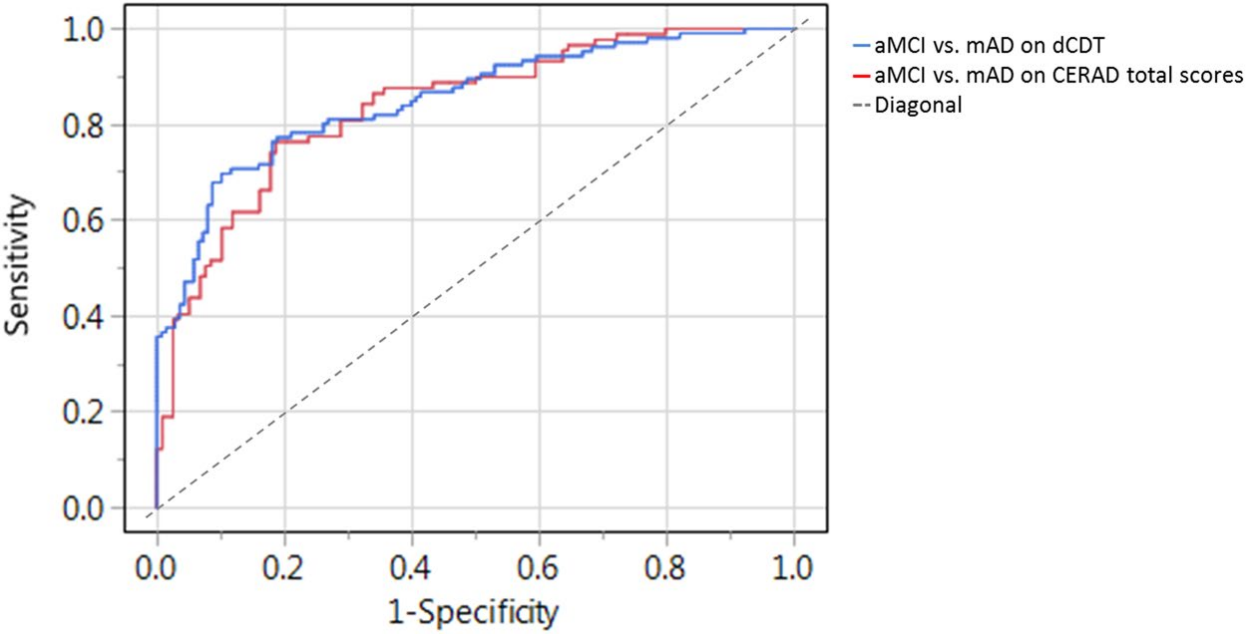
AUCs for aMCI against mAD scores using dCDT Model d (total time, pressure/velocity relation, strokes per minute, and CDT scores; blue curve) or CERAD total scores (red curve).

When predicting patients with aMCI compared with mAD (Table 5, Model d), total time, pressure/velocity relation, strokes per minute, and CDT scores classified 81.2% of the cases correctly (AUC: 0.852; p < 0.0001; Fig. 4; Table 7). Finally, in discriminating patients with aMCI from mAD (Table 5, Model d) individuals, an accuracy of 79.2% was found at a CERAD total score of 58.0 points (AUC: 0.846; p < 0.0001; Fig. 4; Table 7).

## Discussion

The objective of the presented study aimed to assess the potential of the fast and easy to use dCDT in differentiating healthy individuals from patients with mAD and/or aMCI. Recent research using this approach has demonstrated its worthiness in differentiating individuals in the course of AD^15^. As an extension of our previous approach, the full functionality of the tablet software was now used to assess multiple variables during dCDT performance in order to enhance its predictive value. We hypothesize that the inclusion of all available tablet variables or their specific combination might result in a stronger predictive value. The potential of this novel approach is compared against the total score of the time consuming CERAD test battery.

The overall results indicate that dCDT parameters have a large discriminative power, comparable to CERAD total scores, and that the assessment of kinematics during drawing using a digitizing tablet could be a useful method in the clinical setting. It is noteworthy that for completing dCDT, the combination of specific parameters is more relevant for group discrimination than the use of single parameter values as the model-extracted parameters differ with respect to the group to be differentiated. This is in contrast to our recent findings, where the combination of specific dCDT variables (i.e. time in air, time on surface, and total time) did not result in improved distinctness of patients with aMCI against HC what probably be attributed to the lack of specificity of the available variables^15^.

The differentiation between aMCI individuals and healthy controls succeeded on a very good level (i.e. accuracy 81.5%) using the variables time in air and time not painting with CDT scores taking into account. This is slightly better than the observed 77.5% accuracy of the CERAD total score. With the variable time in air the correct classification of healthy individuals from aMCI patients with normal CDT score was at almost the same level (i.e. accuracy 78.0%) and similar to that of the CERAD total score (i.e. accuracy 76.0%). The proportion of aMCI patients with normal CDT was nearly 70% among the aMCI group. These findings indicate that even an individual that succeed in clock drawing might be cognitively impaired but undetected using CDT scores alone. This again underlines that a screening using the conventional paper-pencil based CDT is limited when confronted with MCI individuals^31–33^. However, as has been found here, accuracy increases fundamentally if the entire process during task performance is digitally captured.

Time in air seem to be a distinct characteristic that differs between patients with aMCI and cognitively healthy individuals. This is of particular importance, since in the current study a variety of variables were included in the statistical model to determine whether the addition or combination of multiple variables might improve screening power. However, only time in air, as already seen in our previous studies^15,16^, as well as time not painting revealed the best model fit. Both time not painting and time in air reflect intermissions during the drawing or execution process that are most likely associated with planning and executive functioning^10–12,18,34^. Prolonged in-air trajectories and stagnations while completing the task possibly result from malfunction in frontal and temporo-parietal brain areas which negatively affects decision making and cognitive flexibility and thus interfere with dCDT demands^10–12,35–39^.

Besides this screening approach to differentiate between cognitively normal elderly and aMCI individuals, the clinical need to distinguish aMCI patients from already demented cases is of high importance. By taken CDT scores into account, the dCDT variables total time, pressure/velocity relation, strokes per minute seem to be as highly accurate (i.e. 81.2%) in discriminating patients with aMCI and mAD as the CERAD total score (i.e. accuracy: 79.2%). Interestingly, more variables must be taken into account to distinguish between aMCI individuals and patients with mAD. This might be due to the high overlap of cognitive dysfunction between the two groups of patients, as the transition from MCI to dementia state can not be clearly defined on the basis of cognitive parameters alone. Thus, to enhance discriminative power more variables have to be taken into account.

Taking a closer look on the model selected variables compared to aMCI patients mAD individuals seem to require more time to complete the task (total time) that is associated with more omissions (i.e. few strokes per minute) resulting in impaired CDT scores (e.g. they forgot a clock hand).

In differentiating patients with mAD from healthy individuals both dCDT variables including time in air together with CDT scores (i.e. 93.0% accuracy) and CERAD total scores (i.e. 92.3% accuracy) showed excellent screening power.

In summary, the digital assessment of drawing parameters during dCDT yielded comparable to slightly better screening values for identifying aMCI patients among healthy controls than the use of the CERAD test battery total score. Furthermore, dCDT seems to be a screening instrument of high validity even in aMCI individuals who scored well in conventional CDT. Additionally for clinical diagnostics, dCDT is of high relevance in identifying aMCI patients before these individuals reach the stage of dementia.

Thus, dCDT is an innovative screening tool, particularly to discriminate individuals with slight cognitive disturbances from healthy persons. As its diagnostic performance is at least equal to the elaborated extensive CERAD test, dCDT results could be used as indicator for further diagnostics (e.g., CSF investigation or brain imaging) and could even replace further neuropsychological assessment, especially in the absence of adequate infrastructure (e.g., in rural environment).

With respect to interpretation of the results, potential limitations of this study should be taken into account. Although all participants underwent a training session to get familiar with the use of the stylus and the tablet we did not assessed the habitual use of tablets what might confound the results as most of the variables depend on time. Additionally, increased duration might result from increased effort with the aim to draw the numbers in the clock face carefully. However, this might be probable while drawing the circle whereas writing numbers - in contrast to define their correct position, what causes intermissions and in-air trajectories - is highly automated and therefore unlikely to bias the results. Finally, we can not rule out the possibility that the regression analysis overfit the variables during selection process as a result from the high number of observations included in the statistical model. Unfortunately, cross-validation using a random sample was not carried out to check the results for reliability.

In conclusion, the dCDT offers the unique possibility to track the entire visuo-constructive sequence during clock drawing while multiple variables are captured simultaneously. Nevertheless, it is easy to use by clinicians and the software offers a variety of modifications and can be tailored to the needs of the user. This novel screening technique may be supportive in the early detection and follow-up evaluation of AD-related cognitive disturbances and should be available to a broader clinical setting in the near future.
